# Effect of different types of cerebral perfusion for acute type A aortic dissection undergoing aortic arch procedure, unilateral versus bilateral

**DOI:** 10.1186/s12893-020-00957-8

**Published:** 2020-11-18

**Authors:** Zhengqin Liu, Chen Wang, Xiquan Zhang, Shuming Wu, Changcun Fang, Xinyan Pang

**Affiliations:** 1 Cardiac Surgical Intensive Care Unit, Qilu Hospital of Shandong University, Jinan, 250012 Shandong China; 2 Department of Cardiovascular Surgery, Qilu Hospital of Shandong University, Wenhuaxi Road, Jinan, 250012 Shandong China

**Keywords:** Cerebral protection, Unilateral antegrade cerebral perfusion, Bilateral antegrade cerebral perfusion, Type A aortic dissection, Neurological complications

## Abstract

**Background:**

Antegrade cerebral perfusion (ACP), including unilateral and bilateral, is most commonly used for cerebral protection in aortic surgery. There is still no consensus on the superiority of the two methods. Our research aimed to investigate the clinical effects of u-ACP and b-ACP.

**Methods:**

321 of 356 patients with type A aortic dissection were studied retrospectively. 124 patients (38.6%) received u-ACP, and 197 patients (61.4%) received b-ACP. We compared the incidence of postoperative neurological complications and other collected data between two groups. Besides, we also analyzed perioperative variables to find the potential associated factors for neurological dysfunction (ND).

**Results:**

For u-ACP group, 54 patients (43.5%) had postoperative neurological complications, including 22 patients (17.7%) with permanent neurologic dysfunction (PND) and 32 patients (25.8%) with temporary neurologic dysfunction (TND). For b-ACP group, 47 patients (23.8%) experienced postoperative neurological complications, including 16 patients (8.1%) of PND and 31 patients (15.7%) of TND. The incidence of PND and TND were significantly different between two groups along with shorter CPB time (p = 0.016), higher nasopharyngeal temperature (p≦0.000), shorter ventilation time (p = 0.018), and lower incidence of hypoxia (p = 0.022). Furthermore, multivariate stepwise logistic regression analysis confirmed that preoperative neurological dysfunction (OR = 1.20, p = 0.028), CPB duration (OR = 3.21, p = 0.002), and type of cerebral perfusion (OR = 1.48, p = 0.017) were strongly associated with postoperative ND.

**Conclusions:**

In our study, it was observed that b-ACP procedure exhibited shorter CPB time, milder hypothermia, shorter ventilation time, lower incidence of postoperative hypoxia, and neurological dysfunction compared to u-ACP. Meanwhile, the incidence of ND was independently associated with three factors: preoperative neurological dysfunction, CPB time, and type of cerebral perfusion.

## Background

Aortic dissection (AD) is one of the most serious cardiac emergencies owing to associated high mortality rates, especially Stanford type A [1, 2]. Presently, surgery is the primary treatment method. The frozen elephant trunk technique is increasingly being used to repair dissections extending over the entire aortic arch. Despite immense improvements in surgical techniques, the operative mortality and complications of type A aortic dissection (AD) remain considerably high [3]. Neurological dysfunction (ND) is a common complication, with a reported case rate ranging from 5.5 to 33.3% [4]. Consequently, it is crucial to implement appropriate measures to prevent cerebral injury.

In recent decades, various kinds of protocols to avoid cerebral damage have been utilized. Advances in these protocols have subsequently improved neurological outcomes for type A AD. Currently, antegrade cerebral perfusion (ACP) has become the standard method of cerebral support. However, there is still a controversy regarding the superiority of either the unilateral ACP (u-ACP) or bilateral ACP (b-ACP) [5, 6].

Generally, the feasibility of u-ACP for keeping the brain perfused during the circulatory arrest period is based on the integrity of the circle of Willis. Nevertheless, there are several anatomical variations of the circle. Particularly, b-ACP is an approach of providing cerebral perfusion through both sides simultaneously to mimic the physiological conditions. Therefore, we postulated that b-ACP may be more advantageous for cerebral support or other aspects related to mortality, neurological outcome, and other systemic complications, compared to u-ACP.

We also retrospectively gathered potential variables according to the Society of Thoracic Surgeons (STS) National Database, aiming at identifying risk factors for postoperative neurological dysfunction in patients with type A AD undergoing total aortic arch replacement.

## Methods

### Study populations

We retroactively studied 356 patients with type A AD who were admitted to our cardiac surgical intensive care unit after total aortic arch replacement from January 1, 2014 to December 31, 2018. 321 patients undergoing total arch replacement with one of the following manifestations shown on CTA: arch tear, carotid dissection or occlusion, or the presence of an aortic arch aneurysm were included into our research, of whom 124 underwent a u-ACP (38.6%) and 197 underwent a b-ACP (61.4%). The experimental protocol and informed consent were approved by the Institutional Review Board of our hospital, and all subjects gave informed consent. Among the 321 patients, there were 224 males and 97 females. The mean age was 51.98 ± 9.78 years. All the cases were diagnosed using preoperative computed tomography angiography (CTA) and therapy was conducted within 2 weeks of onset. Preoperative echocardiography was also necessary to evaluate the cardiac function, the exact position of intima rupture, and to assess the condition of the aortic and mitral valves. Every patient underwent total arch replacements. According to the surgery time from onset, we divided patients into three groups: hyper-acute (< 24 h), acute (24–72 h) and subacute (> 72 h). The concomitant procedures included Bentall procedure, coronary artery bypass grafting (CABG) and Bentall + CABG. The relevant demographic data and surgical strategies were illustrated in Table 1. Preoperative neurological complications were defined as acute neurological dysfunctions from dissection, which included one patient with coma, three with somnolence, one with hemiplegia, and four with monoplegia.

**Table 1:** Demographic and preoperative characteristics

Variable	u-ACP (n = 124)	b-ACP (n = 197)	p value
Age (year, $\overline{x}$ ± *s*)	55.38 ± 10.40	54.00 ± 9.37	0.646
Male (n, %)	85 (68.5%)	139 (70.6%)	0.822
BMI (kg/m^2^, $\overline{x}$ ± *s*)	24.58 ± 1.25	25.21 ± 1.44	0.148
Hypertension (n, %)	94 (76.3%)	157 (80.1%)	0.615
Diabetes mellitus (n, %)	16 (12.9%)	43 (21.8%)	0.433
COPD (n, %)	3 (2.4%)	7 (3.6%)	0.746
Cerebrovascular disease history (n, %)	23 (18.5%)	37 (18.8%)	0.958
Renal dysfunction (n, %)^a^	5 (4.0%)	12 (6.1%)	0.423
Hemodynamic instability	4 (3.2%)	9 (4.6%)	0.552
Results of CT scan			
Arch tear	105	152	0.361
Carotid dissection	6	8	
Aortic arch aneurysm	13	37	
Preoperative neurological complication (n, %)	5 (4.0%)	11 (5.6%)	0.534
Preoperative intubation (n, %)	6 (4.8%)	10 (5.1%)	0.666
LVEF < 0.4 (n, %)	4 (3.2%)	10 (5.1%)	0.578

p < 0.05 is considered as statistically significant

*u-ACP* unilateral antegrade cerebral perfusion, *b-ACP* bilateral antegrade cerebral perfusion, *COPD* chronic obstructive pulmonary disease

^a^A serum creatinine level > 97 μmol/L is considered to indicate renal dysfunction

The anesthetic regimen was kept standard. Intravenous (IV) injections of 2.5–5 mg midazolam, 0.2–0.6 mg/kg etomidate, 0.1–5 μg/kg sufentanil, and 0.6 mg/kg rocuronium were performed as the standard anesthetic induction in these patients. Subsequently, tracheal intubations were executed. Anesthesia was maintained by continuous inhalation of sevoflurane, IV injections of dexmedetomidine and rocuronium, combined with theadditional administration of midazolam and sufentanil when necessary.

### Operative technique

All operations were conducted by the same surgical staff. Our operative techniques are composed of cardiopulmonary bypass (CPB), moderate hypothermia (24–28 ℃), circulatory arrest, and ACP. Monitoring data included left radial and dorsalis pedis arterial pressures, main arterial pressure (MAP), central venous pressure (CVP), electrocardiography (ECG), blood oxygen saturation (SaO2), and arterial blood gas (ABG) analysis. Cerebral saturation was monitored with near-infrared spectroscope (NIRS). Prior to December 2015, u-ACP with moderate hypothermia circulatory arrest (24–26 ℃) was routinely performed to maintain cerebral perfusion. From January 2016, b-ACP was initiated until the end of the study. The temperature of circulatory arrest was gradually increased to 26–28 ℃. In both groups, right axillary artery with right femoral artery cannulations were conducted to establish CPB. Besides, cannulation of the right axillary artery was used for cerebral perfusion in the u-ACP group. However, for patients with dissection extending from the innominate artery and into right subclavian, we chose to cut off the right common carotid artery and cannulated directly into the true lumen where dissection was relatively less involved. In the b-ACP group, right axillary artery cannulation was employed along with a 12-F or 14-F balloon-tip catheter in the left common carotid artery.

After CPB was established, we cross-clamped the ascending aorta after the nasopharyngeal temperature dropped to 34 ℃ or lower. Consequently, cold blood cardioplegia was injected into the coronary Ostia antegrade or the coronary sinus retrograde directly to stop the heart. Subsequently, we made a longitudinal incision on the ascending aorta and performed the aortic root procedure depending on the severity and extent of the disease, including aortic root formation or Bentall procedure with or without CABG.

When the temperature reached 24–28 ℃, all three branches of the aortic arch were separately clamped, and the systemic circulation stopped. In the u-ACP group, cerebral perfusion was provided only by the right axillary artery cannulation. Moreover, 12 of 124 patients, who immediately switched to b-ACP, were classified as b-ACP group patients. In the b-ACP group, both the right axillary artery and left common carotid artery were used for cerebral protection. Flow rates of 5–10 ml/kg/min were used for ACP, with a perfusion pressure range of 50–80 mmHg. A stented elephant trunk was inserted into the proximal descending aorta and attached to the distal end of the graft. Subsequently, the systemic circulation of the lower body was restarted through the right femoral artery. For the u-ACP group, anastomosis of the left common carotid artery was first performed to restore left cerebral perfusion. Afterwards, the proximal aortic root was anastomosed to the prosthetic graft to restore systemic circulation, and the temperature increased progressively. The left subclavian artery and innominate artery were sequentially anastomosed to the prosthetic graft. For the b-ACP group, the left subclavian artery was initially anastomosed to the prosthetic graft, followed by the left common carotid artery, proximal aortic root, and innominate artery.

### Statistical analysis

All of the perioperative data were analyzed distinctively between two groups (see Tables 2, 3). The postoperative variables consisted of 30-day mortality rate, ventilation time (h), permanent neurological dysfunction (PND), temporary neurological dysfunction (TND), acute kidney injury (AKI, referring to elevated serum creatinine concentration > 1.5 times of baseline or urine volume < 0.5 ml/kg/h for 6 h within 48 h after surgery), hypoxia (PaO_2_/FiO_2_ < 200 with PEEP ≥ 5cm H_2_O within 72 h after surgery), and postoperative bleeding volume within 24 h of surgery. Patients with neurological symptoms had to be examined by CT scans to confirm the diagnosis. PND was defined as the presence of permanent neurological deficits persisting after being discharged with focal or global cerebral lesions confirmed by CT or MRI, including monoplegia, hemiplegia, paraplegia and coma, confirmed by CT or MRI. TND included transient ischemic attack (TIA) and reversible neurological deficits such as delirium, confusion and agitation with no new lesions on CT. All the diagnoses of stroke were adjudicated by a neurologist who was blinded.

**Table 2:** Intraoperative data

Variable	u-ACP (n = 124)	b-ACP (n = 197)	p
CPB duration (min, $\overline{x}$ ± *s*)	260.07 ± 76.79	235.79 ± 46.60	0.016
Aortic cross-clamping duration (min, $\overline{x}$ ± *s*)	154.53 ± 36.50	154.94 ± 33.75	0.946
Circulatory arrest time (min, $\overline{x}$ ± *s*)	26.60 ± 6.78	25.62 ± 6.31	0.384
Nasopharyngeal temperature (℃, $\overline{x}$ ± *s*)	24.92 ± 0.28	27.09 ± 1.22	0.000
Concomitant procedures (n, %)			
Bentall	25 (20.2%)	47 (23.9%)	0.703
CABG	9 (7.3%)	18 (9.1%)	
Bentall + CABG	7 (5.6%)	13 (6.6%)	
Surgery time (n, %)			
Hyper-acute (< 24 h)	24 (19.4%)	35 (17.8%)	0.914
Acute (24-72 h)	82 (66.1%)	131 (66.5%)	
Subacute (> 72 h)	18 (14.5%)	31 (15.7%)	

p < 0.05 is considered as statistically significant

*u-ACP* unilateral antegrade cerebral perfusion, *b-ACP* bilateral antegrade cerebral perfusion, *CPB* cardiopulmonary bypass, *CABG* coronary artery bypass grafting

**Table 3:** Postoperative data

Variable	u-ACP (n = 124)	b-ACP (n = 197)	p
Drainage volume within 24 h > 1 L (n, %)	22 (17.7%)	27 (13.7%)	0.328
Ventilation time (h, $\overline{x}$ ± *s*)	62.26 ± 46.42	44.03 ± 28.74	0.018
Hpoxia (n, %)	34 (27.4%)	33 (16.8%)	0.022
AKI (n, %)	18 (14.5%)	22 (11.1%)	0.376
PND (n, %)	22 (17.7%)	16 (8.1%)	0.009
TND (n, %)	32 (25.8%)	31 (15.7%)	0.027
ICU stay (days, $\overline{x}$ ± *s*)	18.73 ± 5.67	17.10 ± 5.18	0.447
30-day mortality (n, %)	12 (9.7%)	10 (5.1%)	0.112

p < 0.05 is considered as statistically significant

*u-ACP* unilateral antegrade cerebral perfusion, *b-ACP* bilateral antegrade cerebral perfusion, *AKI* acute kidney injury, *PND* permanent neurologic dysfunction, *TND* temporary neurologic dysfunction

Continuous variables were expressed as mean ± SD; categorical data were expressed as proportions. The T test or Mann–Whitney test were used to compare continuous variables while categorical data were compared using the χ^2^ test or the Fisher exact test. We divided the patients into two groups (ND vs non-ND). All potential risk variables were analyzed for significance. For factors with p values < 0.05, the multivariable logistic regression model was employed to further identify independent risk factors.

## Results

### Intraoperative data

There were no significant differences regarding rates of concomitant surgery, including the Bentall procedure, CABG, and Bentall + CABG between the two groups (Table 2). However, we observed that the CPB durations and nasopharyngeal temperatures during the circulatory arrest period were considerably different.

### Mortality rates and morbidities

After meticulous comparisons of the postoperative data, we did not discover differences in the 30-day mortality rates, incidence of postoperative renal failure, or bleeding volume > 1 L within 24 h of surgery. Compared to the u-ACP group, the patients in b-ACP group exhibited shorter ventilation times (44.03 ± 28.74 vs. 62.26 ± 46.42; p = 0.018) and overall lower incidence of hypoxia (16.4% vs. 27.9%; p = 0.037) (see Table 3).

### Neurological events

**Fig. 1:**
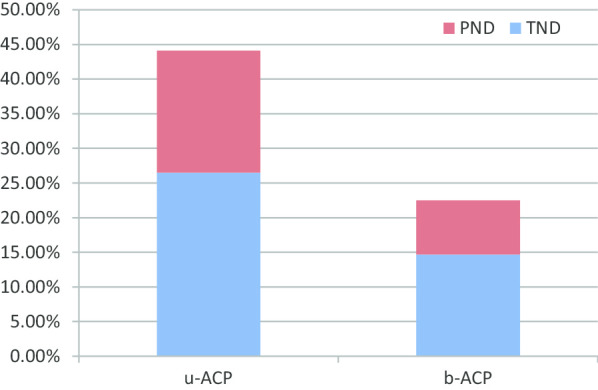
Postoperative neurological dysfunction in the u-ACP and b-ACP groups

We compared the postoperative neurological complications between the two groups (see Fig. 1). For the u-ACP group, PND was observed in 22 patients (17.7%): paraplegia (n = 2), monoplegia (n = 4), hemiplegia (n = 11), and coma (n = 5). TND was observed in 32 patients (25.8%): delirium (n = 23), TIA (n = 2), and confusion and agitation (n = 7). Meanwhile, a total of 16 patients (8.1%) with PND were observed in the b-ACP group: paraplegia (n = 1), hemiplegia (n = 9), monoplegia (n = 4), and coma (n = 2); whilst 31 patients (15.7%) with TND were observed: delirium (n = 25), confusion and agitation (n = 6), and no TIA. The incidence of PND and TND were undoubtedly different between the two groups (see Table 4).

**Table 4:** Description of ND between u-ACP and b-ACP groups

ND	u-ACP (n = 124)	b-ACP (n = 197)
PND	22 (17.7%)	16 (8.1%)
Paraplegia	2 (1.6%)	1 (0.5%)
Monoplegia	4 (3.2%)	4 (2.0%)
Hemiplegia	11 (8.9%)	9 (4.6%)
Coma	5 (4.0%)	2 (1.0%)
TND	32 (25.8%)	31 (15.7%)
Delirium	23 (18.5%)	25 (12.7%)
TIA	2 (1.6%)	0 (0%)
Confusion and agitation	7 (5.6%)	6 (3.0%)

*u-ACP* unilateral antegrade cerebral perfusion, *b-ACP* bilateral antegrade cerebral perfusion, *PND* permanent neurologic dysfunction, *TND* temporary neurologic dysfunction

Furthermore, we divided the patients into different groups based on the incidence of postoperative neurological dysfunction: ND group and non-ND group. The results of univariate analyses of all the potential risk variables were listed in Table 5. All variables with p values < 0.05 were included in the multivariable analysis model. Our study demonstrated that preoperative neurological dysfunction (OR = 1.20, p = 0.028), CPB duration (OR = 3.21, p = 0.002) and type of cerebral perfusion (OR = 1.48, p = 0.017) were independent risk factors for genesis of ND (Table 6).

**Table 5:** Univariate analysis of perioperative risk factors of postoperative ND in patients with type A aortic dissection

Variable	ND (n = 101)	Non-ND (n = 220)	p
Preoperative variables			
Age (year, $\overline{x}$ ± *s*)	56.70 ± 9.91	53.83 ± 9.75	0.422
Male (n, %)	73 (73.7%)	155 (69.8%)	0.475
Education level (year, $\overline{x}$ ± *s*)	10.61 ± 5.30	11.23 ± 4.85	0.651
Tobacco use (n, %)	52 (52.5%)	108 (48.6%)	0.521
Alcohol use (n, %)	20 (20.2%)	37 (16.7%)	0.444
Barrier of hearing or language (n, %)	3 (3.0%)	7 (3.2%)	1.000
Hypertension (n, %)	87 (87.9%)	168 (75.7%)	0.012
Diabetes mellitus (n, %)	23 (23.2%)	36 (16.2%)	0.134
COPD (n, %)	4 (4.0%)	6 (2.7%)	0.505
Cerebrovascular disease history (n, %)	26 (26.3%)	34 (15.3%)	0.020
Peripheral arterial disease (n, %)	7 (7.1%)	11 (5.0%)	0.447
Renal dysfunction (n, %)	9 (9.1%)	8 (3.6%)	0.043
LVEF < 0.4 (n, %)	5 (5.1%)	9 (4.1%)	0.684
Preoperative neurological dysfunction (n, %)	10 (9.9%)	6 (2.7%)	0.005
Preoperative intubation (n, %)	9 (8.9%)	7 (3.2%)	0.024
Preoperative cardiac arrhythmia (n, %)	8 (7.9%)	11 (5%)	0.273
Intraoperative variables			
Concomitant procedures (n, %)			
Bentall	29 (28.7%)	43 (19.5%)	0.358
CABG	15 (14.9%)	12 (5.5%)	
Bentall + CABG	10 (9.9%)	10 (4.5%)	
CPB duration (min, $\overline{x}$ ± *s*)	259.93 ± 69.94	234.48 ± 49.24	0.009
Aortic cross-clamping duration (min, $\overline{x}$ ± *s*)	157.96 ± 37.17	153.25 ± 33.11	0.418
Circulatory arrest time (min, $\overline{x}$ ± *s*)	27.06 ± 6.05	25.33 ± 6.59	0.112
Type of cerebral perfusion (u-ACP, %)	50 (49.5%)	74 (33.6%)	0.007
Nasopharyngeal temperature (℃, $\overline{x}$ ± *s*)	26.11 ± 1.49	26.60 ± 1.38	0.039
Blood transfusion > 1 L (n, %)	25 (24.8%)	34 (15.5%)	0.046
Postoperative variables			
Postoperative drainage volume within 24 h (> 1 L) (n, %)	15 (14.9%)	16 (7.3%)	0.033
AKI (n, %)	16 (15.8%)	25 (11.4%)	0.264
Hemoglobin (g/L, $\overline{x}$ ± *s*)	93.25 ± 10.39	100.50 ± 9.35	0.180
Cardiac arrhythmia (n, %)	21 (50.0%)	36 (19.7%)	0.335

p < 0.05 is considered as statistically significant

**Table 6:** Multivariate stepwise logistic regression analysis for predictors of postoperative ND in patients with type A aortic dissection

Variable	Regression coefficient	OR (95% CI)	p
Preoperative neurological dysfunction^a^	0.391	1.20 (1.07–1.36)	0.028
CPB duration^b^	1.501	3.21 (1.43–5.72)	0.002
Type of cerebral perfusion^b^	0.660	1.48 (1.07–2.04)	0.017

^a^Preoperative variables

^b^Intraoperative variables

## Discussion

We compared the two cerebral perfusion methods concerning a set of clinical outcomes. We discovered that patients in the b-ACP group had shorter CPB times, higher permissive arrest temperatures, shorter ventilation times, and lower incidences of postoperative hypoxia and ND. ND is a serious postoperative complication of acute type A AD [7], with an incidence range of 0–32.8% [8]. Nevertheless, its major risk factors remain uncertain, despite the relatively frequent occurrence of ND in type A AD patients. Therefore, we analyzed various potential factors retrospectively and revealed that the preoperative neurological function, CPB time, and type of cerebral perfusion chosen were independent risk factors for postoperative neurological dysfunction. Consequently, we may reduce the occurrence of postoperative ND by shortening the CPB time and choosing the most efficient cerebral perfusion method. Besides, the relationship between postoperative neurological complications and the intraoperative ACP method used is further discussed in this report.

Hypothermic circulatory arrest combined with ACP has been recognized as the first choice for cerebral protection in aortic arch surgery and was used worldwide [9, 10]. This technique yielded the best results and mostly improved short-term or long-term outcomes. Nevertheless, the use of u-ACP vs b-ACP remains a subject of debate. We routinely used right axillary artery cannulation for u-ACP in type A AD operations. Its main advantage is that it involves less risk of dissection or atherosclerosis in the right axillary artery. Besides, the isolation and cannulation procedure is much easier in this way. Nowadays, along with the tremendous improvements in operative technology, intubation methods, and clearer determination of anatomical locations, a substantial body of evidence suggests the exact effect of b-ACP with advantages of shorter CPB time, permissive milder hypothermia, lower incidence of hypoxia and ND [11, 12]. The possible reasons can be summarized as follows.

We previously explained the surgical procedures with u-ACP or b-ACP in detail. The anastomosis sequence of the three branches of the aortic arch in the two groups is different. For the u-ACP group, the left common carotid artery was initially anastomosed to restore the physiological status of the left cerebral perfusion, followed by the anastomosis of the left subclavian artery. The initial anastomosis of the left common carotid artery can hinder the subsequent operation on the left subclavian artery by reducing the surgical field, making the procedure more challenging and time-consuming. Consequently, the incidence of anastomotic bleeding of the left subclavian artery rose, and our surgeons needed more time and attention to stop the bleeding. The mean CPB time of the u-ACP group was longer than that of the b-ACP group. Although the sequence of anastomosis of supra-arotic branches mostly depended on surgeons' personal choice, we preferred to first anastomose left common carotid artery for patients in the u-ACP group since it provided 2/3 blood-supply of left hemicerebrum. Besides, the sequence of anastomosis of branches mainly depended on the importance of cerebral perfusion. For patients in the b-ACP group, the bilateral cannulation for cerebral perfusion made us more confident to first anastomose the left subclavian artery to make the procedure easier regardless of cerebral blood-supply.

The integrity of the circle of Willis is the key for sufficient cerebral perfusion under u-ACP. Nonetheless, there are several anatomical variations of the circle, not to mention cases of vascular dysplasia or deficiency of vascular branches. Studies have verified that the proportion of individuals with intact circles of Willis comprise 21–25% of the population, and the variation rate can be as high as 50%, especially in the posterior circulation [13, 14]. However, some patients cannot undergo CTA, magnetic resonance angiography (MRA), or digital subtraction angiography (DSA) to determine the integrity of the circle of Willis before surgery due to the severity of the disease itself or for other reasons. Therefore, b-ACP can assure continuous bilateral cerebral perfusion regardless of the integrity of the circle of Willis. Harrer et al. [15] reported that the left cerebral oxygen saturation increased by about 19% when the perfusion approach was switched from u-ACP to b-ACP.

With the advent of deep hypothermic circulatory arrest (DHAC) into clinical practice by Griepp and associates in the 1970s, the outcome of arch surgery was remarkably improved. Hypothermia provides neurological protection essentially by decreasing the global cerebral metabolic rate and increasing the cerebral tolerance to circulatory arrest. Hypothermia can also reduce temperature-dependent release and extracellular levels of excitatory neurotransmitters such as glutamate, contributing to inhibiting pro-apoptotic activity and reducing the level of free radicals and inflammatory cytokines [16]. Earlier protocols have utilized temperatures as low as 14℃ with the belief that lower temperature can sufficiently diminish cerebral metabolic demands. In recent years, surgeons began to question the limitations of DHAC. Ehrlich et al. reported that further reduction of temperature beyond 18℃ did not further decrease the oxygen requirement in the brain. It led to greater cerebrovascular homeostasis impairment and reduced cerebral perfusion [17]. Several experts have cautioned against DHAC because of its related hazards of prolonged CPB time, greater coagulopathy, and aggravated inflammatory responses. Recent studies from high-volume aortic centres have demonstrated excellent results of moderate levels of hypothermia combined with ACP [18, 19]. With the increase in the circulatory arrest temperature, the tolerance of cerebral tissue to ischemia or hypoxia reduced. In the b-ACP group, both cerebral hemispheres were perfused directly through cannulation, permitting better perfusion and a higher arrest temperature compared with the u-ACP group. Particularly, the higher circulatory arrest temperature caused less impaired cerebrovascular homeostasis, avoiding hypo-cerebral or hyper-cerebral perfusion through the physiological regulation of the cerebral vessels themselves. Finally, the higher temperature during circulatory arrest enabled shorter cooling and rewarming time, substantially reducing the CPB time.

### Study limitations

This study has some inevitable limitations. First, the study is a retrospective review for 5 years, and we cannot exclude the influence of era bias. Second, this study only represents a single-centre experience instead of a multicenter experience. Thus, we need to design a multicenter prospective analysis in the future to further verify our results.

## Conclusions

The b-ACP procedure had some advantages compared to u-ACP, including reduced incidence of postoperative neurological dysfunction, shorter ventilation time, shorter CBP time, and lower incidence of postoperative hypoxia, according to our research. Furthermore, we analyzed the potential risk factors for ND and revealed that preoperative neurological dysfunction, CPB duration, and perfusion method were independent risk factors for the generation of ND. Therefore, we may reduce the incidence of ND by modifying the adjustable factors including CPB time and more appropriate cerebral perfusion method.

## Data Availability

The datasets analysed during the current study are available from the corresponding author on reasonable request.

## Abbreviations

AD: Aortic dissection
ND: Neurological dysfunction
ACP: Antegrade cerebral perfusion
u-ACP: Unilateral antegrade cerebral perfusion
b-ACP: Bilateral antegrade cerebral perfusion
CPB: Cardiopulmonary bypass
MAP: Mean arterial pressure
CVP: Central venous pressure
ECG: Electrocardiography
SaO_2_: Blood oxygen saturation
ABG: Arterial blood gas
NIRS: Near-infrared spectroscope
PND: Permanent neurological dysfunction
TND: Temporary neurological dysfunction
AKI: Acute kidney injury
TIA: Transient ischemic attack
DHAC: Deep hypothermic circulatory arrest
